# Paclitaxel-etoposide-carboplatin/cisplatin versus etoposide-carboplatin/cisplatin as first-line treatment for combined small-cell lung cancer: a retrospective analysis of 62 cases

**DOI:** 10.7497/j.issn.2095-3941.2015.0012

**Published:** 2015-06

**Authors:** Yue-Ya Li, Chan Zhou, Deng-Xia Yang, Jing Wang, Zhu-Jun Liu, Xin-Yue Wang, Kai Li

**Affiliations:** Department of Thoracic Oncology, Tianjin Medical University Cancer Institute and Hospital, National Clinical Research Center for Cancer, Key Laboratory of Cancer Prevention and Therapy, Tianjin Lung Cancer Diagnosis and Treatment Center, Tianjin 300060, China

**Keywords:** Small cell lung carcinoma (SCLC), chemotherapy, CE regimen, adverse effects, survival analysis, prognosis

## Abstract

**Objective:**

To compare the efficacy and adverse effects of paclitaxel-etoposide-carboplatin/cisplatin (TEP/TCE) regimen with those of etoposide-carboplatin/cisplatin (EP/CE) regimen as first-line treatment for combined small-cell lung cancer (CSCLC).

**Methods:**

A retrospective study was conducted on 62 CSCLC patients who were treated at Tianjin Medical University Cancer Institute and Hospital from July 2000 to April 2013 and administered with TEP/TCE regimen (*n*=19) or EP/CE regimen (*n*=43) as first-line CSCLC treatment. All patients received more than two cycles of chemotherapy, and the response was evaluated every two cycles. The primary endpoint was overall survival (OS), and the secondary endpoints were progression-free survival (PFS), objective response rate (ORR), disease control rate (DCR), and adverse effects.

**Results:**

ORR between the TEP/TCE and EP/CE groups showed a statistical difference (90% *vs*. 53%, *P*=0.033). Both groups failed to reach a statistical difference in DCR (100% *vs*. 86%, *P*=0.212). The median PFS and OS of the TEP/TCE group were slightly longer than those of the EP/CE group, although both groups failed to reach a statistical difference (10.5 *vs*. 8.9 months, *P*=0.484; 24.0 *vs*. 17.5 months, *P*=0.457). However, stratified analysis indicated that the PFS of patients with stages III and IV CSCLC showed marginally significant difference between the TEP/TCE and EP/CE groups (19.5 *vs*. 7.6 months; *P*=0.071). Both rates of grade IV bone marrow depression and termination of chemotherapy in the TEP/TCE group were significantly higher than those in the EP/CE group (26.3% *vs*. 7.0%, *P*=0.036; 31.6% *vs*. 14.7%, *P*=0.004).

**Conclusion:**

The TEP/TCE regimen may not be preferred for CSCLC, and this three-drug regimen requires further exploration and research. To date, the EP/CE regimen remains the standard treatment for CSCLC patients.

## Introduction

Small-cell lung cancer (SCLC) accounts for approximately 20% of all lung cancers worldwide^1^^,^^2^. Combined small-cell lung cancer (CSCLC) is a subtype of SCLC, representing 2%-28% of SCLC cases^3^^,^^4^. According to the 2004 World Health Organization (WHO)/International Association for the Study of Lung Cancer classification of lung and pleural tumors^5^, CSCLC is defined as SCLC combined with non-small cell lung cancer (NSCLC) components, which usually include adenocarcinoma (Ad), squamous-cell carcinoma, large-cell carcinoma^6^^-^^8^, and spindle cell carcinoma^9^^,^^10^. Despite the rare incidence of CSCLC, this malignancy not only grows fast but also resists chemotherapy. Thus, this cancer is taken seriously in clinical studies. Unfortunately, no standard regimen has been determined for CSCLC; therefore, its treatment mainly refers to the therapeutic regimens of pure SCLC, such as etoposide-carboplatin/cisplatin (EP/CE)^8^. However, CSCLC often yields poor prognosis because the combined NSCLC components may be insensitive to such chemotherapy regimens. Given that the majority of combined components of CSCLC were Ad^8^^,^^10^^,^^11^.Zhu *et al*.^12^ added paclitaxel to the EP/CE regimen, thereby forming paclitaxel-etoposide-carboplatin/cisplatin (TEP/TCE) regimen for CSCLC. Nevertheless, a consensus on whether the efficiency and security of TEP/TCE regimen is superior to the standard EP/CE regimen remains unclear. The present retrospective study included 62 CSCLC patients, who were diagnosed pathologically. These patients underwent complete follow-up sessions and received initial treatment at the Tianjin Medical University Cancer Institute and Hospital from July 2006 to April 2013. On the basis of the chemotherapy regimens administrated, 62 patients were classified into two-drug group (receiving EP/CE regimen) and three-drug group (receiving TCE/TEP regimen) to compare the tumor response, survival benefits, and adverse effects of the two groups.

## Materials and methods

### Eligibility of patients

A total of 62 primary CSCLC patients who were treated at the Cancer Hospital of Tianjin Medical University from July 2006 to April 2013 were enrolled in this study. The following inclusion criteria were applied: (I) patients were diagnosed with CSCLC, which was confirmed via pathology or cytology; (II) patients were previously naive to chemotherapy, radiotherapy, or surgery; (III) patients exhibited no other malignancies; (IV) the lesions of patients can be evaluated via imaging; (V) their ages ranged from 34-79, and Karnofsky Performance Status (KPS) score was ≥60; (VI) the results of their blood, routine urine, electrolyte, liver function, kidney function, and electrocardiogram tests were within normal range; (VII) patients underwent complete follow-up sessions.

### Chemotherapy

The two regimens were TEP/TCE (paclitaxel 135 mg/m^2^, intravenous on day 1; etoposide 100 mg/m^2^, intravenous on days 1-3; carboplatin calculated at the area under the curve (AUC) =5, intravenous on day 1 or cisplatin 25 mg/m^2^, intravenous on days 1-3) and EP/CE (etoposide 100 mg/m^2^, intravenous on days 1-3; carboplatin calculated at AUC =5, intravenous on day 1 or cisplatin 25 mg/m^2^, intravenous on days 1-3).

### Evaluation of response

CT or MRI scan was performed to evaluate tumor response every two chemotherapeutic cycles and at the end of treatment. Patients were examined monthly within 3 months after the end of treatment, every 2 months within 1 year after the end of treatment, and every 3-6 months thereafter. Unidirectional measurements were conducted in accordance with the Response Evaluation Criteria in Solid Tumors (RECIST) version 1.1 to evaluate short-term effects. Following the RECIST1.1, tumor response to treatment was classified as complete response (CR), partial response (PR), stable disease (SD), and progressive disease (PD).

Objective response rate (ORR) was defined as the proportion of patients evaluated as CR and PR, whereas disease control rate (DCR) was calculated as the proportion of patients evaluated as CR, PR, and SD. Overall survival (OS) was defined as the time from the start of treatment until death caused by any cause or until the last follow-up date. Moreover, progression-free survival (PFS) was defined as the time from the start of treatment to disease progression or death.

### Toxicity

Chemotherapy-related adverse reactions were divided into five degrees (0-IV) based on the WHO classification of acute and subacute toxicity performance and indexing standards.

### Statistical analysis

Statistical analyses were performed with SPSS 13.0. A *P* value ≤0.05 was considered statistically significant. Kaplan-Meier method was used to estimate the PFS and OS. Multivariate analysis of the prognostic factors was performed using Cox’s regression model. Categorical variables were analyzed via χ^2^ test, and measurement data were analyzed using *t*-test.

## Results

A total of 540 CSCLC patients existed in 2,371 SCLC cases; thus, the incidence of CSCLC was 22.78%. Finally, we collected the data of 62 CSCLC patients who have met our inclusion criteria. Among 62 CSCLC patients, 49 were males and 13 were females, and the ratio of male patients to female patients was 3.85:1. The age of patients ranged from 34-79 years old, and the median age was 60. Smoking history was confirmed in 51 cases. In accordance with TNM staging, 5 patients were categorized in stage I, 5 patients were in stage II, and 52 patients were in advanced stage (25 patients were in stage III and 27 patients were in stage IV). The two chemotherapy regimen groups showed no significant differences in the baseline data (**Table 1**).

**Table 1:** General condition of 62 primary CSCLC patients, *n* (%)

Characteristics	EP/CE group	TEP/TCE group	χ^2^	*P*
Age (years)			0.433	0.551
>60	22 (51.2)	8 (42.1)		
≤60	21 (48.8)	11 (57.9)		
Gender			0.000	0.991
Male	34 (79.1)	15 (78.9)		
Female	9 (20.9)	4 (21.1)		
Smoking index			0.587	0.444
>400	25 (58.1)	13 (68.4)		
≤400	18 (41.9)	6 (31.6)		
KPS			2.935	0.087
>80	37 (86.1)	19 (100.0)		
≤80	6 (3.9)	0 (0.0)		
Stage			2.492	0.571
I	4 (9.3)	1 (5.3)		
II	3 (7.0)	2 (10.5)		
III	15 (34.9)	10 (52.6)		
IV	21 (48.8)	6 (31.6)		
No. of chemotherapy cycles			6.173	0.103
1-2 cycles	5 (11.6)	7 (36.8)		
3-4 cycles	15 (34.9)	3 (15.8)		
5-6 cycles	19 (44.2)	7 (36.8)		
>6 cycles	4 (9.3)	2 (10.5)		
First-line chemotherapy			1.897	0.168
Platinum				
Cisplatin	19 (44.2)	12 (63.2)		
Carboplatin	24 (55.8)	7 (36.8)		
Chest radiotherapy	12 (27.9)	5 (26.3)	0.017	0.897
Prophylactic cranial radiotherapy (PCI)	8 (18.6)	1 (5.3)	1.890	0.169
Surgical resection	9 (20.9)	6 (31.6)	0.815	0.367
Second-line chemotherapy			3.217	0.522
No treatment	4 (14.3)	3 (42.9)		
IP regime	8 (28.6)	1 (14.3)		
The original regimen	6 (21.4)	1 (14.3)		
TP regimen	7 (25.0)	1 (14.3)		
Local radiotherapy	3 (10.7)	1 (14.3)		
Third-line chemotherapy	8 (18.6)	2 (10.5)	0.636	0.425

Smoking index: (number of cigarettes smoked per day) × years. CSCLC, combined small-cell lung cancer; KPS, Karnofsky Performance Status; IP regimen, irinotecan plus platinum; TP regimen, paclitaxel plus platinum.

A total of 15 patients at stages I, II, and IIIa (T_1-3_N_2_M_0_) received surgery and adjuvant chemotherapy with 3-drug regimen or 2-drug regimen. Other patients received radiation combined with chemotherapy or chemotherapy alone. Among the 62 patients, 19 received TEP/TCE regimen and 43 received EP/CE regimen.

Both chemotherapy regimens were administered at an interval of 3 weeks, and each patient completed at least 2 cycles of chemotherapy. Some patients accepted thoracic radiotherapy within 2 to 4 cycles of chemotherapy with a total dose of 50 Gy, which was administered with 2 Gy per fraction and conducted 5 d a week. Some other patients received prophylactic cranial irradiation with a total dose of 30 Gy, which was administered with 3 Gy per fraction and conducted 5 d a week after the chemotherapy was completed.

### Effects

The TEP/TCE and EP/CE groups showed a statistical difference in ORR (90% *vs.* 53%, *P*=0.033, χ^2^=4.552). However, both groups failed to reach a statistical difference in DCR (100% *vs.* 86%, *P*=0.212, χ^2^=1.558) (**Table 2**).

**Table 2:** Comparison of response between two groups, *n* (%)

Group	CR	PR	SD	PD	ORR	DCR	*P*
EP/CE	0 (0.0)	19 (52.8)	12 (33.3)	5 (13.9)	19 (52.8)	31 (81.6)	0.235
TEP/TCE	0 (0.0)	9 (90.0)	1 (10.0)	0 (0.0)	9 (90.0)	10 (100)	

ORR = CR + PR; DCR = CR + PR + SD. CR, complete response; PR, partial response; SD, stable disease; PD, progressive disease; ORR, objective response rate; DCR, disease control rate.

### Survival analysis

**Figure 1:**
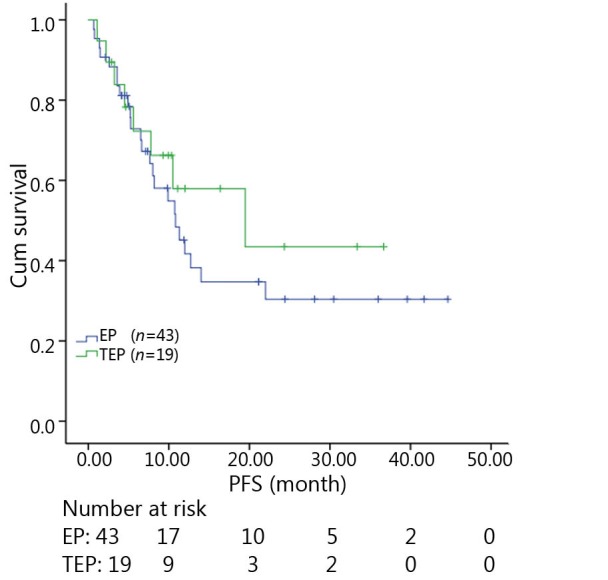
Kaplan-Meier curves for PFS of 62 patients with stages I-IV CSCLC. The median PFS of 3-drug group was not significantly longer than that of 2-drug group (10.5 *vs*. 9.8 months, *P*=0.484). PFS, progression-free survival; CSCLC, combined small-cell lung cancer.

**Figure 2:**
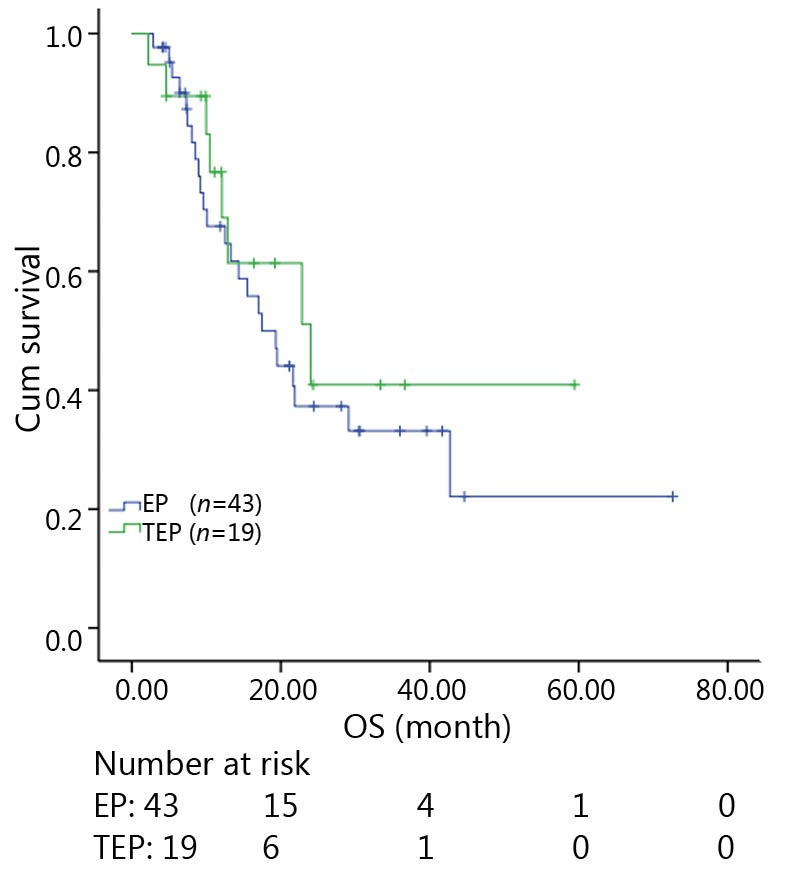
Kaplan-Meier curves for OS of 62 patients with stages I-IV CSCLC. The median OS of 3-drug group was not significantly longer than that of 2-drug group (24.0 *vs*. 17.5 months, *P*=0.457). OS, overall survival; CSCLC, combined small-cell lung cancer.

**Figure 3:**
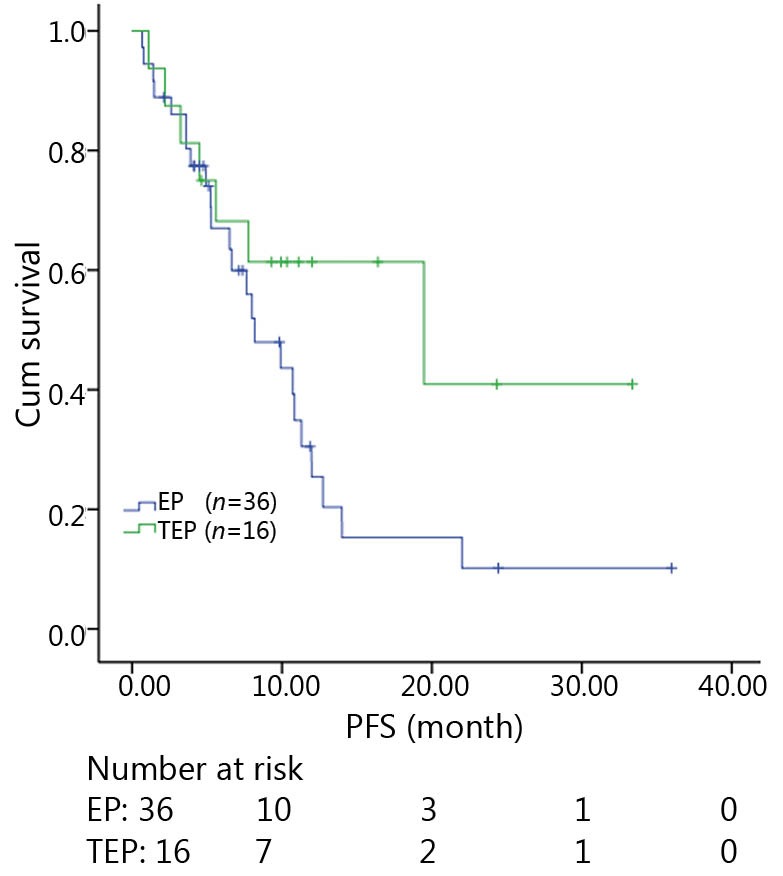
Kaplan-Meier curves for PFS of 52 patients with stages III-IV CSCLC. The median PFS of the two groups nearly reached a significant difference (19.5 *vs*. 7.6 months, *P*=0.071). PFS, progression-free survival; CSCLC, combined small-cell lung cancer.

**Figure 4:**
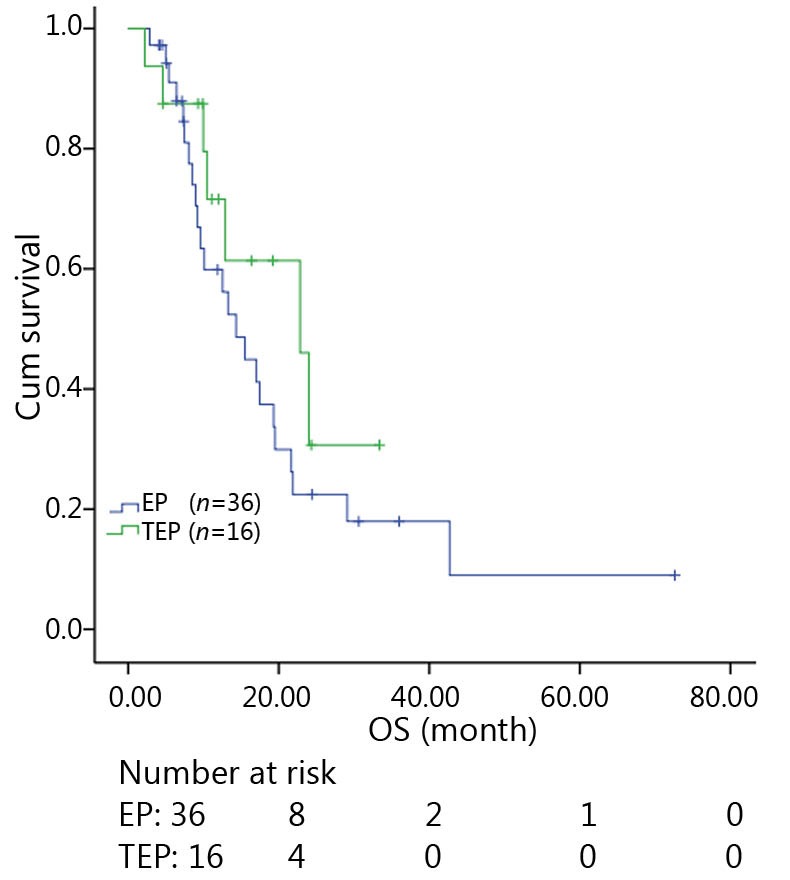
Kaplan-Meier curves for OS of 52 patients with stages III-IV CSCLC. The difference in median OS between the two groups was marginally significant (22.8 *vs*. 14.3 months, *P*=0.269). OS, Overall survival; CSCLC, Combined small-cell lung cancer.

**Figure 5:**
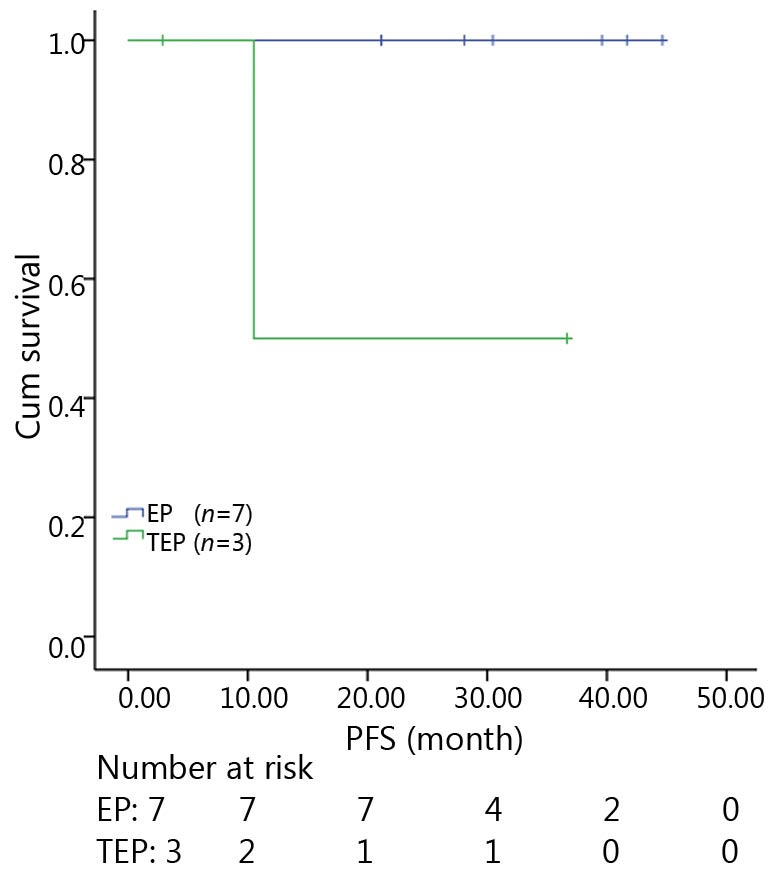
Kaplan-Meier curves for PFS of 10 patients with stages I-II CSCLC. The median PFS of 3-drug group was not significantly longer than that of 2-drug group (10.5 *vs*. 30.5 months, *P*=0.061). PFS, progression-free survival; CSCLC, combined small-cell lung cancer.

**Figure 6:**
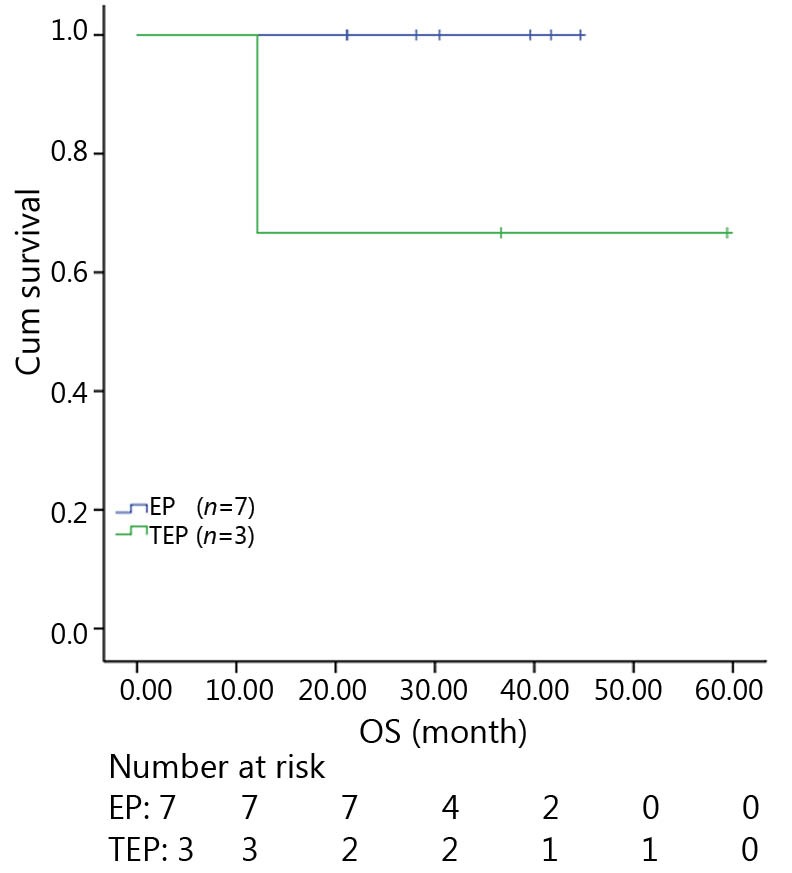
Kaplan-Meier curves for OS of 10 patients with stages I-II CSCLC. The median OS of 3-drug group was not significantly longer than that of 2-drug group (36.7 *vs*. 30.5 months, *P*=0.127). OS, overall survival; CSCLC, combined small-cell lung cancer.

All patients were followed up until November 28, 2013, and the median follow-up time was 12.7 months (range, 2-73 months). A total of 30 patients were alive at the end of follow-up, which comprised 11 patients from the TEP/TCE group and 19 patients from the EP/CE group. The median PFS and OS of the TEP/TCE group were slightly longer than those of the EP/CE group, although both groups failed to reach a statistical difference (10.5 *vs.* 9.8 months, *P*=0.484, χ^2^=0.489; 24 *vs.* 17.5 months, *P*=0.457, χ^2^=0.554) (**Figures 1****,****2**). However, stratified analysis indicated that in patients with stages III and IV CSCLC, the median PFS nearly reached a statistical difference between the TEP/TCE and EP/CE groups (19.5 *vs.* 7.6 months, *P*=0.071, χ^2^=3.259), whereas the median OS failed to reach a statistical difference (22.8 *vs.* 14.3 months, *P*=0.269, χ^2^=1.224) (**Figures 3****,****4**). However, no significant difference existed between the two groups at stages I and II (**Figures 5****,****6**).

### Univariate analysis

The results showed that patients with KPS score ≤80, distant metastasis, lymph node metastasis, and tumors at stages III and IV displayed poor prognosis (*P*<0.001, *P*=0.005, 0.032, and 0.001, respectively). Furthermore, the absence of surgery and prophylactic cranial irradiation (PCI) were considered adverse prognostic factors (*P*=0.009, 0.042). However, age, gender, smoking index, tumor size, number of chemotherapy cycles, thoracic radiotherapy, second-/third-line therapy were not related to the prognosis (**Table 3**).

**Table 3:** Cox regression analysis of the factors affecting the survival of 62 patients

Characteristics	*n*	MST (month)	χ^2^	*P*	
				Univariate	Multivariate
Age (years)			1.086	0.297	
>60	30	19.5			
≤60	32	21.9			
PS (scores)			18.298	<0.001	0.015
>80	56	21.87			
≤80	6	7.47			
Gender			0.173	0.678	
Male	49	19.5			
Female	13	19.3			
Smoking index			1.183	0.277	
>400	38	17.0			
≤400	24	21.9			
Tumor size (cm)			1.478	0.224	
>3	44	17.5			
≤3	18	42.7			
Lymph node metastasis			4.589	0.032	0.007
Yes	49	14.3			
No	13	NR			
Distant metastasis			7.781	0.005	0.007
Yes	26	42.7			
No	36	10.1			
Stage			10.980	0.001	
I-II	10	NR			
III-IV	52	17.0			
No. of chemotherapy cycles			2.593	0.459	
1-2 cycles	12	12.1			
3-4 cycles	18	21.7			
5-6 cycles	26	19.5			
>6 cycles	6	NR			
Chest radiotherapy			0.514	0.473	
Yes	17	21.9			
No	45				
Prophylactic cranial irradiation			4.141	0.042	0.034
Yes	9	42.7			
No	53	15.5			
Surgical resection			6.889	0.009	
Yes	15	NR			
No	47	15.5			
Second-line chemotherapy			5.477	0.242	
No treatment	7	12.1			
IP regimen	9	9.2			
The original regimen	7	19.3			
TP regimen	8	21.9			
Local radiotherapy	4	12.5			
Third-line chemotherapy			1.297	0.255	
Yes	10	17.5			
No	52	19.5			

Smoking index, (number of cigarettes smoked per day) × years; NR, not reached; KPS: Karnofsky Performance Status; IP regimen, irinotecan plus platinum; TP regimen, paclitaxel plus platinum; MST, median survival time.

### Multivariate analysis

A multivariate analysis of prognostic factors was performed using Cox’s regression model. The results showed that the KPS score, lymph node metastasis, distant metastasis, and PCI were independent predictors of prognosis (*P*=0.015, 0.007, 0.007, and 0.034, respectively) (**Table 3**).

### Safety evaluation

The common chemotherapy-related adverse reactions included bone marrow suppression, gastrointestinal reaction, hepatic and renal function lesions, and skin rash. Most of these reactions were slight and reversible. The rates of grade IV bone marrow depression in the TEP/TCE group were significantly higher than those in the EP/CE group (26.3% *vs.* 7.0%, *P*=0.036, χ^2^=4.385). However, although the incidences of skin rash and diarrhea were higher in the TEP/TCE group than in the EP/CE group, both groups (10.5% *vs.* 0%, *P*=0.09, χ^2^=4.677) displayed no statistical difference. The statistical data showed that the replacement and incompletion of chemotherapy were more prevalent in the TEP/TCE group than in the EP/CE group because of serious adverse reactions (31.6% *vs.* 4.7%, *P*=0.004, χ^2^=8.502), although the main reason for replacement and incompletion of chemotherapy in the EP/CE group was the disease progression (32.6% *vs.* 5.3%, *P*=0.021, χ^2^﹦5.353).

## Discussion

CSCLC is currently defined by the WHO as a subset of SCLC, which not only exhibits the characteristics of small cell lung cancer, such as rapid growth and high malignant degree, but also displays the characteristics of NSCLC, such as chemotherapy resistance. Patients with CSCLC have demonstrated poor response to chemotherapy in previous studies^13^^,^^14^. Researchers attributed this finding to the combined NSCLC components. Thus, they attempted to explore ideal chemotherapy regimens. Luo *et al*.^8^ compared the efficacy and safety of vinorelbine, ifosfamide, and cisplatin (NIP) with EP in treating advanced CSCLC, and they concluded that the ORR, PFS, and OS of patients in the NIP group were slightly inferior than those of patients under traditional EP regimen (83.8% *vs.* 90.6%, *P*=0.170; 6 *vs.* 6.5 months, *P*=0.163; 10.8 *vs.* 10.4 months, *P*=0.935, respectively). The TEP/TCE regimen has been widely used for CSCLC treatment at the Tianjin Medical University Cancer Institute and Hospital since 2005, with the aim of increasing the dose intensity and coverage rate of antitumor spectrum. The incidence of CSCLC in this institution was 22.78%, which was consistent with the findings of previous reports^3^^,^^4^. In our study, a large proportion of patients were male (79.03%), heavy smokers (61.29%), and beyond 60 years old, which also corresponded to the study conducted by Lu *et al*.^15^.

The ORRs of the TEP/TCE and EP/CE groups were 90% and 53%, respectively, which reached a significant difference (*P*=0.033, χ^2^=4.552). Furthermore, the DCRs of the two groups indicated no significant difference (100% *vs.* 86%, *P*=0.212, χ^2^=1.558). The PFS and OS of the patients in the TEP/TCE group were both slightly longer than those of the patients in the EP/CE group (11.86 *vs.* 12.14 months; 17.65 *vs.* 18.01 months, respectively). However, both groups failed to reach a statistical difference. Moreover, safety analysis showed that the incidence of grade IV bone marrow depression and grades III and IV diarrhea was significantly higher in the TEP/TCE group than in the EP/CE group (*P*=0.004). Further analysis revealed that such adverse reactions in the TEP/TCE group were nearly consistent with the toxicity of paclitaxel. Statistical data revealed that the replacement and incompletion of chemotherapy were more prevalent in the TEP/TCE group than in the EP/CE group because of serious adverse effects (31.6% *vs.* 4.7%, *P*=0.04, χ^2^=8.502). In summary, the administration of TEP/TCE regimen can provide beneficial short-term effects, but such effects cannot prolong the PFS and OS of patients with CSCLC. Multivariate analysis also confirmed that only the KPS score, lymph node metastasis, distant metastasis, and PCI were independent prognostic factors for patients with CSCLC. However, subgroup analysis of patients at stages III and IV revealed that the PFS and OS of patients treated with TEP/TCE regimen are slightly longer than those of patients treated with EP/CE regimen. Moreover, the differences nearly reached statistical significance (10.95 *vs.* 8.20 months, *P*=0.071; 10.2 *vs.* 17.62 months, *P*=0.089, respectively). This finding suggested that the TEP/TCE regimen may be beneficial to patients with advanced CSCLC and large tumor burden. The statistical data also showed that the main reason of replacement and completion of chemotherapy in the EP/CE group was disease progression (32.6% *vs.* 5.3%, *P*=0.021, χ^2^=5.353). Hence, broad-spectrum antitumor regimen may be superior to standard EP/CE regimen. However, making reasonable choices on the added drugs and reducing the incidence of side reactions are urgent problems that need to be solved. Ad is the most common combined component of CSCLC^8^^,^^10^^,^^11^ and the effectiveness rate of paclitaxel combined with cisplatin in the treatment of NSCLC (including Ad) reached 22%-47%^16^^-^^19^. Although taxane is good for NSCLC, whether this drug induces the same effect on NSCLC components of CSCLC is unknown. Wagner *et al*.^7^ determined that the NSCLC and SCLC components of CSCLC shared an identical immunophenotype with prevalent expression of synaptophysin and CD56 and loss of 22q13. Fukui *et al*.^20^ also determined that patients with CSCLC with Ad shared an identical EGFR mutation in both SCLC and Ad components. Thus, the current findings suggested that NSCLC components of CSCLC may be close to SCLC in biology, and two kinds of components may exhibit the homology of gene sequence. We used next-generation sequencing method to compare and analyze whether the gene expression of Ad components in CSCLC is different from NSCLC. We believe that the results of our study will clarify the correlation of SCLC components with NSCLC components in CSCLC and contribute to the selection of the optimal treatment for CSCLC.

In conclusion, EP/CE regimen remains the standard regimen for majority of patients with CSCLC. However, three-drug regimen may increase the curative effect of patients in advanced stage. Nevertheless, this investigation is a retrospective, small, and nonrandomized study. Thus, the results need to be further confirmed by large prospective clinical trials.
